# Toxicity and efficacy of three dose-fractionation regimens of intensity-modulated radiation therapy for localized prostate cancer

**DOI:** 10.1093/jrr/rrt124

**Published:** 2013-10-17

**Authors:** Yoshihiko Manabe, Yuta Shibamoto, Chikao Sugie, Fumiya Baba, Shiho Ayakawa, Aiko Nagai, Shinya Takemoto, Akihiro Hayashi, Noriyasu Kawai, Mitsuru Takeuchi, Satoshi Ishikura, Kenjiro Kohri, Takeshi Yanagi

**Affiliations:** 1 Department of Radiology, Nagoya City University Graduate School of Medical Sciences, 1 Kawasumi, Mizuho-cho, Mizuho-ku, Nagoya 467-8601, Japan; 2 Department of Radiation Oncology, West Medical Center, 1-1-1 Hirate-cho, Kita-ku, Nagoya 462-8508, Japan; 3 Department of Radiology, Social Insurance Chukyo Hospital, 1-1-10 Sanjo,Minami-ku, Nagoya 457-8510, Japan; 4 Department of Urology, Nagoya City University Graduate School of Medical Sciences, 1 Kawasumi, Mizuho-cho, Mizuho-ku, Nagoya 467-8601, Japan; 5 Department of Radiation Oncology, Juntendo University Faculty of Medicine, 2-1-1 Hongo, Bunkyo-ku, Tokyo 113-8421, Japan

**Keywords:** prostate cancer, intensity-modulated radiation therapy, hypofractionated radiation therapy

## Abstract

Outcomes of three protocols of intensity-modulated radiation therapy (IMRT) for localized prostate cancer were evaluated. A total of 259 patients treated with 5-field IMRT between 2005 and 2011 were analyzed. First, 74 patients were treated with a daily fraction of 2.0 Gy to a total of 74 Gy (low risk) or 78 Gy (intermediate or high risk). Then, 101 patients were treated with a 2.1-Gy daily fraction to 73.5 or 77.7 Gy. More recently, 84 patients were treated with a 2.2-Gy fraction to 72.6 or 74.8 Gy. The median patient age was 70 years (range, 54–82) and the follow-up period for living patients was 47 months (range, 18–97). Androgen deprivation therapy was given according to patient risk. The overall and biochemical failure-free survival rates were, respectively, 96 and 82% at 6 years in the 2.0-Gy group, 99 and 96% at 4 years in the 2.1-Gy group, and 99 and 96% at 2 years in the 2.2-Gy group. The biochemical failure-free rate for high-risk patients in all groups was 89% at 4 years. Incidences of Grade ≥2 acute genitourinary toxicities were 9.5% in the 2.0-Gy group, 18% in the 2.1-Gy group, and 15% in the 2.2-Gy group (*P* = 0.29). Cumulative incidences of Grade ≥2 late gastrointestinal toxicity were 13% in the 2.0-Gy group at 6 years, 12% in the 2.1-Gy group at 4 years, and 3.7% in the 2.2-Gy group at 2 years (*P* = 0.23). So far, this stepwise shortening of treatment periods seems to be successful.

## INTRODUCTION

High-dose (>70 Gy) external beam radiation therapy has been shown to improve local control rates of localized prostate cancer [1, 2]. To obtain high local control rates while maintaining normal tissue toxicities at acceptable levels, intensity-modulated radiation therapy (IMRT) is now used worldwide in the treatment of prostate cancer [3–10]. IMRT has also become popular in Japan, but because of its relatively short history, published results of IMRT for Japanese patients remain scarce [3–6].

One important issue with IMRT for prostate cancer is the long treatment period; IMRT with 1.8- or 2-Gy daily fractions takes nearly two months or longer, and this constitutes a disadvantage of IMRT compared with brachytherapy. To address this disadvantage, shortening of the treatment period is now being investigated by many groups. The α/β ratio of prostate cancer is presumed to be equivalent to or even lower than the ratio for late-responding normal tissues [11–14], so the use of daily doses higher than 2 Gy may not jeopardize and may even improve the clinical outcome. Some hypofractionated trials using IMRT or 3D radiation therapy have shown outcomes comparable to conventional fractionated radiation therapy [15, 16]. Nevertheless, clinical studies of dose fractionation schedules using equivalent doses calculated by the linear-quadratic (LQ) model are limited [15]. In our institution, we started IMRT in 2004. We first used 2.0-Gy daily fractions to a total dose of 78 or 74 Gy for localized prostate cancer. After evaluating middle-term toxicities, we then tested 2.1-Gy and 2.2-Gy daily doses. The total dose was determined based on the equivalent biological effective dose assuming an α/β ratio of 3 Gy (BED_3_) for late-responding normal tissues. It has been reported that in general the α/β ratio for prostate cancer is somewhat smaller than 3 Gy, [11–14] and the ratio for late rectal bleeding is slightly higher [17, 18]. If these reports are correct, the use of an α/β ratio of 3 Gy would be safe for our attempt at fractional dose escalation. In the present study we have evaluated 6-year results for the 2.0-Gy protocol, 4-year results for the 2.1-Gy protocol and 2-year, mainly toxicity, results for the 2.2-Gy protocol.

## MATERIALS AND METHODS

### Patient characteristics

Between January 2005 and October 2011, 270 patients were treated with a 5-field IMRT technique at Nagoya City University Hospital. Protocols were approved by the institutional review board, and written informed consent was obtained from all patients. The dose-fractionation protocols were revised twice. First, a daily fraction of 2.0 Gy was used up to a total dose of 74 Gy (low risk) or 78 Gy (intermediate or high risk). After evaluating the 12-month toxicities in 40 patients, the daily fraction was increased to 2.1 Gy. The total dose was 73.5 Gy for low-risk patients and 77.7 Gy for intermediate- or high-risk patients. Furthermore, after evaluating the toxicities in 60 patients, the daily dose was again increased to 2.2 Gy. The total dose was 72.6 or 74.8 Gy. These total doses were equivalent in terms of BED_3_ (123–126 Gy for the low-risk patients, and 130–132 Gy for the intermediate- or high-risk patients). After accruing the patient numbers (40 or 60), the same protocols were used until the follow-up periods for the last patients became 12 months. Among the patients seen during the period, 11 were excluded because of long pretreatment androgen deprivation therapy (ADT) for 2 years or more (*n* = 8), or prescription of a different dose because of a patient's wishes or conditions (*n* = 3). Thus, a total of 74 patients treated with 2.0-Gy daily fractions, 101 patients treated with 2.1 Gy daily fractions and 84 patients treated with 2.2 Gy daily fractions were analyzed. The median age of all patients and the follow-up period of living patients were 70 years (54–82) and 47 months (18–97), respectively. The patient characteristics are summarized in Table 1. Patients were staged according to the 7th edition of TNM staging at clinical diagnosis and D'Amico Risk Categories [19], typically using computed tomography (CT), magnetic resonance imaging (MRI) and bone scintigram. All patients had a histologic diagnosis of prostate adenocarcinoma confirmed by pathologists.

**Table 1.** Patient characteristics

Group	All patients	2.0 Gy/day	2.1 Gy/day	2.2 Gy/day	*P-*value
Total dose (Gy)	72.6–74/74.8–78^a^	74/78^a^	73.5/77.7^a^	72.6/74.8^a^	
No. of patients	259	74	101	84	
Age (years)	54–82	54–80	56–80	56–82	0.05^b^
(median)	70	68	70	70	
Initial PSA (ng/ml)	2.6–283	3.2–283	4.6–241	2.6–248	0.91^b^
(median)	11.3	10.8	11.5	10.8	
Risk Low/Intermediate/High	28/82/149	11/19/44	14/31/56	3/32/49	0.09^c^
T Stage 1/2/3	73/110/76	24/30/20	27/44/30	22/36/26	0.91^c^
ADT	224 (86%)	51 (69%)	91 (90%)	82 (98%)	<0.0001^c^
Use of anticoagulant	44 (17%)	15 (20%)	15 (15%)	14 (17%)	0.64^c^
Coexistent DM	43 (17%)	15 (20%)	11 (11%)	17 (20%)	0.14^c^
Follow-up^d^ (months)	18–97	20–97	22–68	18–38	
(median)	47	79	49	27	

PSA = prostate-specific antigen, ADT = androgen deprivation therapy, DM = diabetes mellitus. ^a^For low risk/intermediate or high risk. ^b^Examined by one-factor analysis of variance. ^c^Examined by chi-squared test. ^d^For living patients.

### IMRT and androgen deprivation therapy

For treatment planning, all patients were immobilized in a supine position with a vacuum bag system (BodyFIX; Medical Intelligence, Schwabmuenchen, Germany) for their whole body, and CT scans were performed at a slice thickness of 3.2 mm using a 4-row multidetector CT (Mx8000; Philips Medical Systems, Best, the Netherlands) in a supine position under normal breathing as described in detail previously [20]. CT images were reconstructed at 2.5-mm thickness. The outlines of the target were delineated on a 3D radiation treatment planning system (Eclipse Version 7.5.14.3; Varian Medical Systems, Palo Alto, CA) by reference to images of MRI. The clinical target volume (CTV) included the prostate and seminal vesicles (SV). The CTV of the SV depended on the T stage of the patient: proximal one-third volume of the SV for T1; proximal half of the SV for T2; and the whole SV for T3. We defined the planning target volume (PTV) margin for the CTV to be 8 mm in the anterior, 6 mm in the posterior, 8 mm in the craniocaudal, and 7 mm in the lateral directions. The rectum was contoured from 10 mm below to 10 mm above the PTV in the craniocaudal direction. The prescribed dose represented that mean dose to the PTV. Dose constraints in all groups are summarized in Table 2. For all group patients, the dose constraints were determined by equivalent BED_3_. When the dose constraints could not be fulfilled, the rectal dose constraints took priority. Before treatment of all daily fractions, patients underwent positioning correction using an optically guided 3D-ultrasound target localization system (SonArray, Zmed Inc., Ashland, MA). They were then treated using 18-MV X-rays of a linear accelerator (CLINAC 23EX; Varian Medical Systems, Palo Alto, CA) with five static ports (45, 98, 180, 262 and 315 degrees using dynamic multileaf collimators).

**Table 2.** Dose constrains for the three dose groups

	2.0 Gy/day		2.1 Gy/day		2.2 Gy/day	
Total dose (Gy)	74	78	73.5	77.7	72.6	74.8
PTV						
D95	≥90% of total dose					
V90%	≥96% of total dose					
Mean	≥99% and ≤103% of total dose					
Max	≤110% of total dose					
Rectum Vx						
≤35%	40	40	38	40	37.4	38.5
≤18%	60	60	57	60	56	57.7
=0%	74	78	74	78	72.9	75.1
Bladder Vx						
≤50%	40	40	38	40	37.4	38.5
≤25%	65	65	62	65	60.7	62.5
=0%	74	78	74	78	72.9	75.1

PTV = planning target volume, D95 = minimum dose delivered to 95% of the PTV, V90% = percentage of the PTV receiving at least 90% of the prescribed dose, Vx = percentage of the organ receiving at least X Gy.

It has been our policy to give neoadjuvant ADT for 6 months for intermediate- or high-risk patients, and also adjuvant ADT to high-risk patients for 2–3 years. However, the use and period of ADT were finally determined by consensus with the patients. Neoadjuvant ADT was given to intermediate- or high-risk patients (86% of all patients). Adjuvant ADT was also given to about half the high-risk patients (31% of all patients). The proportion of patients undergoing ADT was lower in the 2.0-Gy group (69%) than in the other groups (90 and 98%, intermediate- and high-risk patients, respectively, *P* < 0.0001).

### Follow-up and data collection

Follow-up evaluations after treatment were performed at 1–3-month intervals until 1 year, and every 3–6 months thereafter. Prostate-specific antigen (PSA) failure was defined as a PSA rise of ≥ 2 ng/ml above the nadir [21]. All endpoints were calculated from the start of IMRT. Toxicities were evaluated with the Common Terminology Criteria for Adverse Events version 4.0. Acute and late toxicities were defined as those occurring within 3 months of starting IMRT, and as those occurring later than 3 months, respectively.

### Statistical analysis

Differences in patient characteristics and incidences of acute genitourinary (GU)/gastrointestinal (GI) toxicities between groups were examined by one-factor analysis of variance and the chi-squared test. Overall survival rates, PSA-failure-free survival (PSA-FFS) rates and cumulative incidences of Grade ≥ 2 late GU/GI toxicity were calculated by the Kaplan–Meier method, and differences between groups were examined by logrank test. Statistical analyses were carried out with the statistical software package ‘R’/package = survival [22].

## RESULTS

**Fig. 1.**
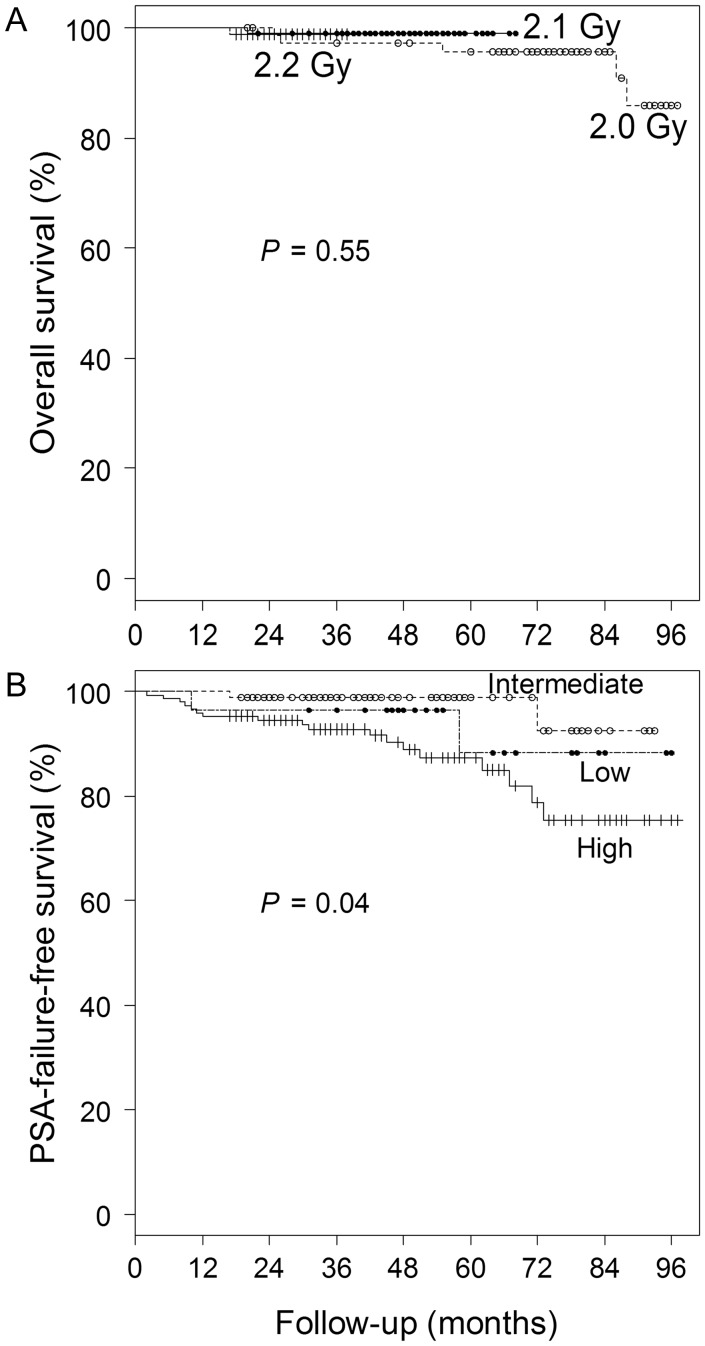
Curves for overall survival in the three dose groups (**A**) and PSA-failure-free survival in the three risk groups (**B**).

**Fig. 2.**
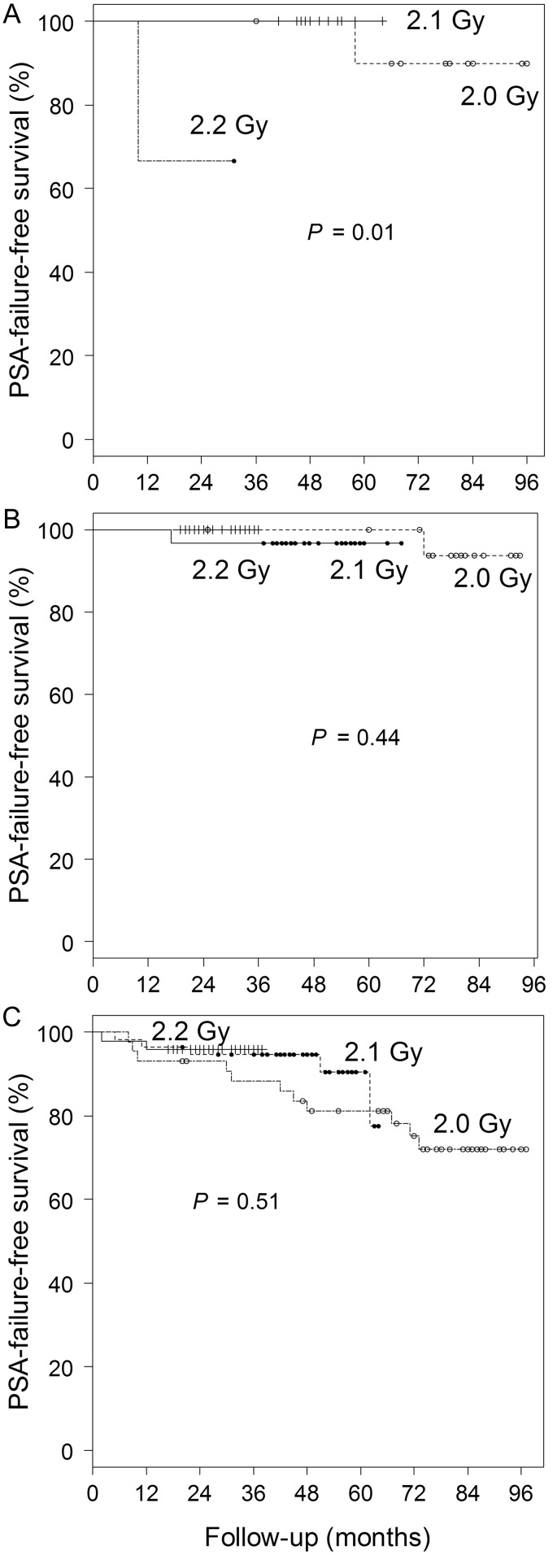
PSA-failure-free survival curves for the three dose groups in low- (**A**), intermediate- (**B**) and high- (**C**) risk patients.

Overall survival and PSA-FFS rates were 96 and 82%, respectively, at 6 years for the 2.0-Gy group, 99 and 96%, respectively, at 4 years for the 2.1-Gy group, and 99 and 96%, respectively, at 2 years for the 2.2-Gy group (Fig. 1). The PSA-FFS rate for high-risk patients in all groups was 89% at 4 years, while those for intermediate- and low-risk patients were 99 and 96%, respectively (*P* = 0.04 for the three groups) (Fig. 1). The number of low-risk patients was only three in the 2.2-Gy group, and one developed PSA failure, so the PSA-FFS rate was 67% at 2 years in the group (Fig. 2). The other groups had good PSA-FFS rates for low-risk patients (90% at 6 years in the 2.0-Gy group and 100% at 4 years in the 2.1-Gy group, Fig. 2). For intermediate- and high-risk patients, the PSA-FFS rates were 94 and 75%, respectively, at 6 years in the 2.0-Gy group, 97 and 95%, respectively, at 4 years in the 2.1-Gy group, and 100 and 96%, respectively, at 2 years in the 2.2-Gy group (*P* = 0.44 and 0.51, respectively, Fig. 2). Only one patient died of prostate cancer.

**Fig. 3.**
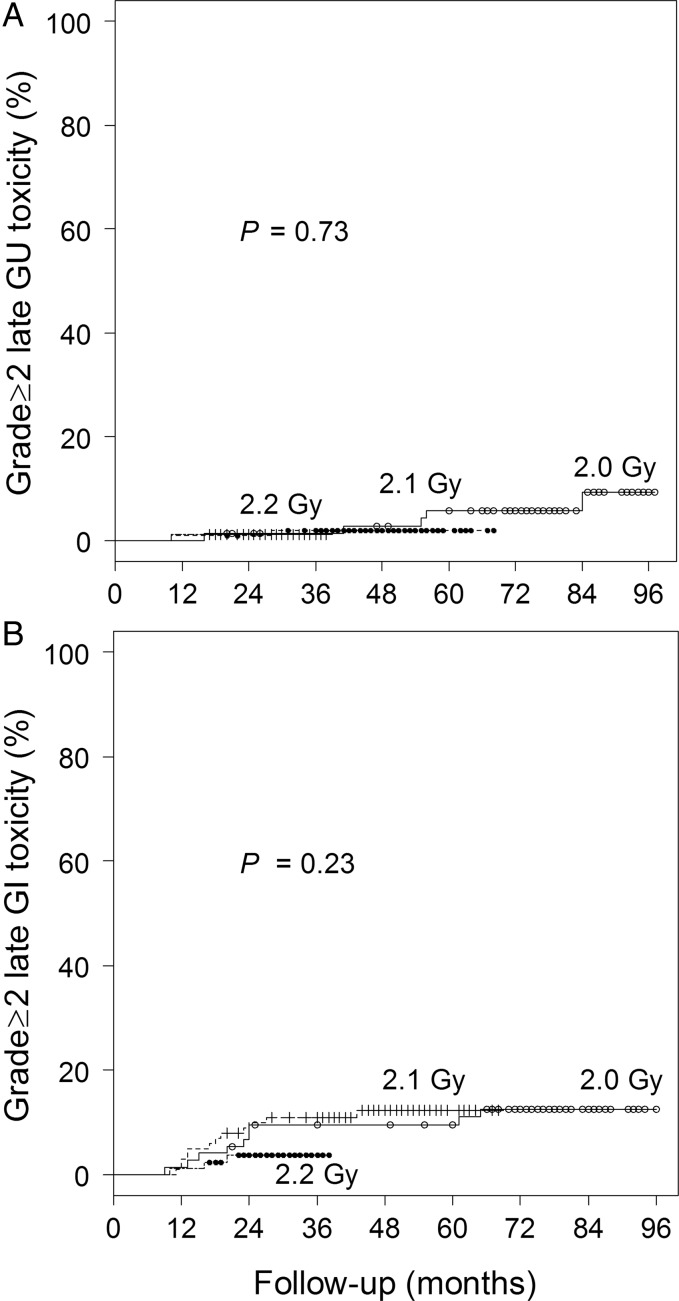
Curves for cumulative incidence of Grade ≥2 late genitourinary (GU) toxicity (**A**) and gastrointestinal (GI) toxicity (**B**) for the three dose groups.

**Fig. 4.**
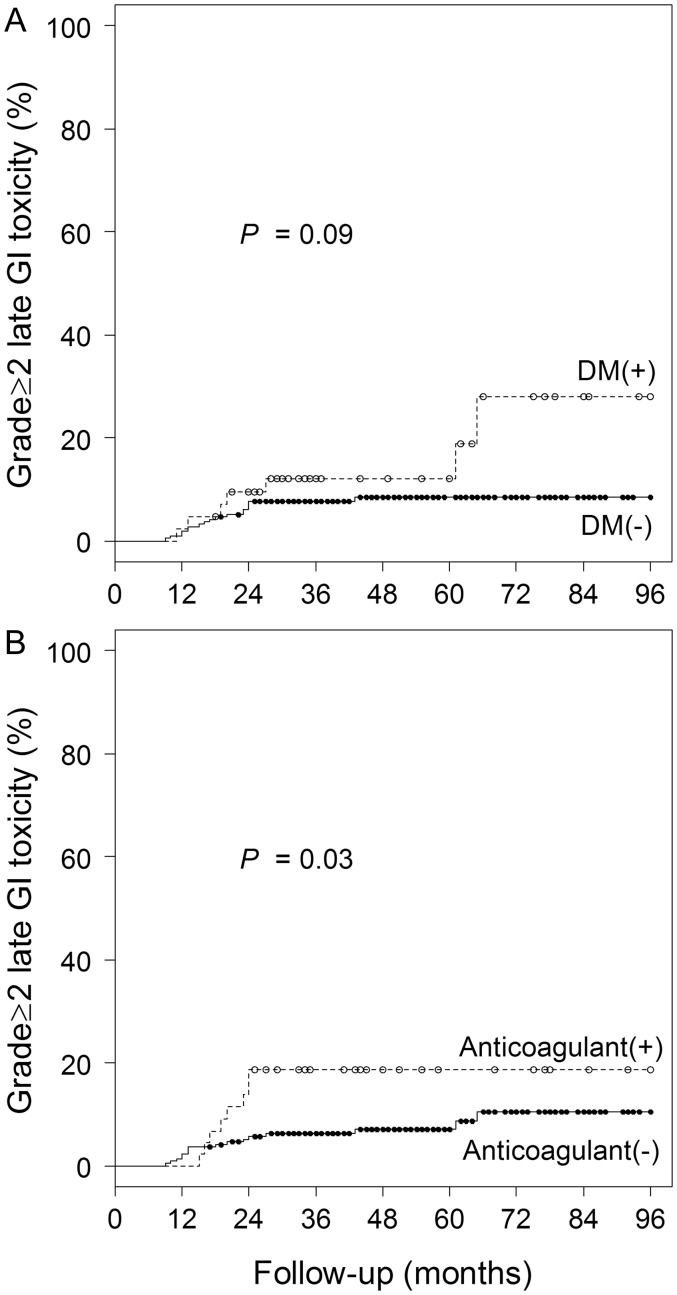
Curves for cumulative incidence of Grade ≥2 gastrointestinal (GI) toxicity in the patients with diabetes mellitus (DM) (**A**) and in those taking anticoagulant (**B**).

The incidences of Grade 2 acute GU and GI toxicity were 9.5 and 1.4%, respectively, for the 2.0-Gy group, 18 and 4.0%, respectively, for the 2.1-Gy group, and 15 and 0%, respectively, for the 2.2-Gy group (*P* = 0.29 and 0.14, respectively, Table 3). No Grade ≥ 3 acute toxicity was observed. Cumulative incidences of Grade ≥ 2 late GU and GI toxicity were 5.8 and 13%, respectively, at 6 years for the 2.0-Gy group, 2.0 and 12%, respectively, at 4 years for the 2.1-Gy group, and 1.2 and 3.7%, respectively, at 2 years for the 2.2-Gy group (*P* = 0.73 and 0.23, respectively) (Table 4, Fig. 3). Argon plasma coagulation (APC) was administered to 15 patients (5.8% of all patients) for rectal hemorrhage. Our technique for APC has been described in detail previously [7]. Two patients (0.77% of all patients) had Grade 3 GI toxicity; rectal hemorrhage developed at 12 and 27 months, respectively. In both patients, the effect of a steroid suppository was limited. Blood transfusion and APC were performed. Anemia due to chronic kidney disease, hemorrhoid and diabetes mellitus coexisted in one of them. Two (0.77% of all patients) had Grade 3 GU toxicity; one with a history of urethrotomy and transurethral resection of the prostate for benign prostatic hyperplasia had urinary tract obstruction due to hematuria at 28 months. It was difficult to stop bleeding despite continuous bladder irrigation. Endoscopic coagulation and blood infusion were required. The other had urinary retention at 24 months and was treated with medication, but urinary tract obstruction with unilateral hydronephrosis developed at 41 months. Improvement was obtained by conservative treatment including urethral catheterization. No patients had Grade ≥ 4 toxicity. There was a significant difference in the incidence of GI toxicity due to the use of anticoagulants (*P* = 0.03), but presence of diabetes mellitus was not a significant factor (*P* = 0.09) (Fig. 4).

**Table 3.** Grade 2 acute toxicity

	2.0 Gy/day	2.1 Gy/day	2.2 Gy/day	*P*-value^a^
Genitourinary				
Urinary frequency (%)	8.1	16	15	0.29
Urinary retention (%)	2.7	2.0	0	0.35
Total (%)	9.5^b^	18	15	0.29
Gastrointestinal				
Rectal hemorrhage (%)	1.4	4.0	0	0.14
Total (%)	1.4	4.0	0	0.14

^a^Examined by chi-squared test. ^b^One patient had both Grade 2 urinary frequency and urinary retention.

**Table 4.** Grade ≥2 late toxicity

	2.0 Gy/day		2.1 Gy/day		2.2 Gy/day	*P*-value
	Grade 2	Grade 3	Grade 2	Grade 3	Grade 2	
Genitourinary						
Urinary frequency (Onset, months)	1 (56)					
Hematuria (Onset, months)	2 (16, 84)		1 (10)	1 (28)		
Urinary retention (Onset, months)		1 (41)			1 (10)	
Urinary incontinence (Onset, months)	1 (55)					
Total^a^	5.8%^b^		2.0%^c^		1.2%^d^	0.73^e^
Gastrointestinal						
Rectal hemorrhage (Onset, median; range, months)	9 (23; 9–65)		10 (18; 11–43)	2 (12, 27)	3 (16; 10–20)	
Total^a^	13%^b^		12%^c^		3.7%^d^	0.23^e^

^a^Cumulative incidence of Grade ≥2 late genitourinary/gastrointestinal toxicity. ^b^Incidence at 6 years. ^c^Incidence at 4 years. ^d^Incidence at 2 years. ^e^Examined by logrank test.

## DISCUSSION

There were no differences in acute and late toxicities among the three groups in the present study. Survival and PSA-failure-free rates also did not seem to differ, although the follow-up periods differed between the three groups. These results indicate that our attempt at shortening the overall treatment time has been successful so far. We assumed an α/β ratio of 3 Gy for BED calculation, but even when an α/β ratio of 1.5 or 4 Gy was used, the three protocols had similar BEDs, so these results may not be surprising. After evaluating longer-term results in the 2.2-Gy group, we plan to move to the next step, i.e. to use 2.3- or 2.4-Gy per fraction.

All groups obtained favorable overall survival and PSA-FFS rates, although the follow-up for the 2.2 Gy group was still short and the proportions of patients undergoing ADT differed between the three groups. Regarding GU toxicity, Zelefsky *et al.* [8] reported that the incidence of Grade 2 GU toxicities increased with radiation dose, from 8% with 70.2 Gy to 20% with 81 Gy, using IMRT. In their study, which had a median follow-up of 10 years, Grade 3 GU toxicity developed in 3% of all patients. The rate of acute Grade ≥ 2 GU toxicity (9.5–18%) and Grade 3 GU toxicity (0.77%, developed at 28 and 41 months) in all patients of this study seemed to compare favorably. However, they also reported a higher incidence and a later onset of urinary Grade 2 toxicity after conformal radiation therapy; the median time to development of Grade ≥ 2 GU symptoms was 30 months, compared with 17 months for patients with Grade ≥ 2 GI toxicity. Thus, longer follow-up would be warranted to determine late Grade ≥ 2 GU toxicity.

The cumulative incidence of Grade ≥ 2 late GI toxicity (8.5% at 3 years in all patients) was comparable with that reported previously [8, 9]. The 2.0-Gy and 2.1-Gy groups had relatively higher rates (13% at 6 years and 12% at 4 years, respectively). This seems to be partly related to our policy of treating rectal bleeding early [7]. It is possible that some cases of bleeding that might stop without treatment were treated; rectal bleeding for which a steroid suppository was prescribed, even in the early stage of bleeding, was classified as Grade 2. The trend towards lower Grade ≥ 2 late GI toxicity in the 2.2-Gy group (3.7% at 2 years) may in part result from an improvement in planning skill. The current study suggested that the use of anticoagulants might be a risk factor for late GI toxicity. This and other risk factors such as the presence of diabetes mellitus and high-dose irradiated volume of the rectum have been reported [23–25]. In the present study, diabetes mellitus was not a risk factor for Grade ≥ 2 GI toxicity, but this might be due to the small patient number. Data derived from 3D-comformal radiation therapy experiences recommended rectal V60 < 35% and V70 < 20% as a conservative starting point for the dose–volume constraints in 3D-comformal radiation therapy [26]; all patients in the present study fulfilled those dose constraints. Tucker *et al.* [23] investigated the risk factors using multivariate analysis of V5 through V85 in 1009 patients treated with Radiation Therapy Oncology Group protocol 94–06, and found that V75 was the only risk factor. However, the rate of Grade ≥ 2 late rectal toxicity was estimated to be 8.9% in the patients treated with 68.4 Gy of total dose, although their V75 was 0. Thus, they hypothesized that there was a ‘background’ level of 8–10%, depending on some biological factors and genetic differences among patients.

Recently, more hypofractionated IMRT or stereotactic body radiation therapy (SBRT) is being investigated for prostate cancer [12, 27]. Miralbell *et al.* [11] analyzed nearly 6000 patients and the calculated α/β ratio was 1.4 Gy. If such a low α/β ratio is true, hypofractionated schedules will increase the therapeutic gain [12]. In estimating the α/β ratio of tumors, however, the reoxygenation phenomenon has never been taken into account, so the α/β ratios estimated by such methods should be interpreted cautiously. Kupelian *et al.* [27] treated 770 consecutive patients with ultrasound-guided IMRT using 2.5-Gy daily fractionation to 70 Gy over 5 weeks and obtained favorable outcomes including tumor control and toxicities. Considering the development and success of SBRT for various cancers [28–30] and favorable results for hypofractionated high-dose-rate brachytherapy [31, 32], SBRT might be an alternative treatment to conventional IMRT for prostate cancer [33–37]. Promising middle-term results have been reported recently [33]. However, the optimal fractionation and total dose should be determined carefully, because the LQ model overestimates the effect of a high fractional dose of radiation [38, 39]; it may only be applicable to fractional doses up to twice the α/β ratio [39]. According to this theory, our approach for stepwise shortening of the overall treatment time based on the LQ model may be reasonable, and it has yielded expected outcomes so far. To further evaluate the advantages of hypofractionation over normofractionated radiation therapy, the results of ongoing randomized trials are awaited [12].

In conclusion, tumor control was good and toxicities were acceptable in all dose groups so far, suggesting that stepwise shortening of treatment periods has been successful.
